# GLYNAC SUPPLEMENTATION IN OLDER ADULTS PROTECTS FROM MEAL DRIVEN OXIDATIVE STRESS AND INFLAMMATION: RESULTS OF A RCT

**DOI:** 10.1093/geroni/igac059.2933

**Published:** 2022-12-20

**Authors:** Rajagopal Sekhar, George Taffet, Premranjan Kumar

**Affiliations:** Baylor College of Medicine, Houston, Texas, United States; Baylor College of Medicine, Houston, Texas, United States; Baylor College of Medicine, Houston, Texas, United States

## Abstract

Elevated oxidative stress (OxS) and inflammation are linked to many age-associated abnormalities, but underlying contributing factors are not well understood. In a randomized clinical trial (RCT) in older adults (OA), we investigated the contribution of a single carbohydrate-meal on OxS and inflammation, and tested the protective effect of supplementing GlyNAC (combination of glycine+N-acetylcysteine) from the meal-driven rise in OxS and inflammation. We studied 20 OA and 10 YA (young-adults) to (1) compare the impact of a 75g oral glucose-meal on plasma markers of OxS (as TBARS) and inflammation (as IL-6); (2) re-assess these outcomes after supplementing OA with either GlyNAC or isonitrogenous placebo (alanine) for 16-weeks, and YA with GlyNAC for 2-weeks. We found that (1) 2-hours after a glucose-meal, compared to YA the OA had higher increases (expressed as percent-change over baseline) of OxS (TBARS –0.1 +/- 0.6 vs. 5.6 +/- 0.6, p < 0.0000) and inflammation (IL-6 3.5 +/- 1.2 vs. 11.2 +/- 0.7, p < 0.0000); (2) GlyNAC supplementation in OA protected against the glucose meal-driven increase in OxS (TBARS pre vs post: 4.8 +/- 0.9 vs. -0.4 +/- 0.8, p=0.002) and inflammation (IL-6 pre vs. post: 10.8 +/- 3.2 vs. 4.2 +/- 2.3, p=0.0001). No improvements occurred in OA receiving placebo or YA receiving GlyNAC. These findings suggest that: (1) In OA, a single carbohydrate-meal significantly increases OxS and inflammation; (2) GlyNAC supplementation protects from carbohydrate meal-driven increases in OxS and inflammation. Additional research to investigate the implications of supplementing GlyNAC on improving meal-related health in aging are warranted.

